# Modelling Motility: The Mathematics of Spermatozoa

**DOI:** 10.3389/fcell.2021.710825

**Published:** 2021-07-20

**Authors:** Eamonn A. Gaffney, Kenta Ishimoto, Benjamin J. Walker

**Affiliations:** ^1^Wolfson Centre for Mathematical Biology, Mathematical Institute, University of Oxford, Oxford, United Kingdom; ^2^Research Institute for Mathematical Sciences, Kyoto University, Kyoto, Japan

**Keywords:** sperm, flagellum, mechanics, modelling, computer-assisted beat-pattern analysis

## Abstract

In one of the first examples of how mechanics can inform axonemal mechanism, Machin's study in the 1950s highlighted that observations of sperm motility cannot be explained by molecular motors in the cell membrane, but would instead require motors distributed along the flagellum. Ever since, mechanics and hydrodynamics have been recognised as important in explaining the dynamics, regulation, and guidance of sperm. More recently, the digitisation of sperm videomicroscopy, coupled with numerous modelling and methodological advances, has been bringing forth a new era of scientific discovery in this field. In this review, we survey these advances before highlighting the opportunities that have been generated for both recent research and the development of further open questions, in terms of the detailed characterisation of the sperm flagellum beat and its mechanics, together with the associated impact on cell behaviour. In particular, diverse examples are explored within this theme, ranging from how collective behaviours emerge from individual cell responses, including how these responses are impacted by the local microenvironment, to the integration of separate advances in the fields of flagellar analysis and flagellar mechanics.

## 1. Observation and theory of sperm motility: an introduction

The fundamental function of a spermatozoon is the fertilisation of an egg in spite of tremendous challenges, whether that be the hostile environments and barriers of the female reproductive tract for internal fertilisers, or harsh osmotic conditions and background fluid flows for external fertilisers. Sufficient motility is thus a core functional necessity of the sperm cell and the attention of extensive study. However, although sperm motility due to a beating flagellum was first observed by van Leeuwenhoek in the 1670s (Lonergan, 2018), the internal structure of the sperm flagellum was only revealed with the advent of electron microscopy, with studies beginning in the 1950s (Fawcett and Porter, 1954; Afzelius, 1959). Even with this methodological step change, it was not at all clear at the time how the complex flagellar structure underlay the mechanism that drives sperm motility. Indeed, this significantly preceded the elucidation of the underlying mechanism of motility, via sliding microtubule filaments driven by dynein molecular motors along the flagellum, which was conclusively demonstrated by Summers and Gibbons (1971). Nonetheless, Machin (1958)'s theoretical study demonstrated that the active processes driving the flagellum could not solely be due to forcing in the cell membrane, reasoning that the wave amplitude of the elastic flagellum would be too damped by drag, even in a low-viscosity fluid such as a water-based physiological electrolyte. As such, Machin's theoretical study was among the first to highlight the importance of mechanics, and its quantification, in understanding how sperm swim.

More generally, the need for a mechanical perspective on the swimming of spermatozoa was recognised in the 1950s, with initial application to sea urchin sperm based on microscopic imaging (Gray and Hancock, 1955). These pioneer studies have been extended and generalised in numerous directions over the past six to seven decades, with recent refinement in particular driven by improvements in the digital microscopy of the flagellum beat and increased computational power, overcoming many of the technological limitations of previous studies. In particular, after briefly summarising classical computational techniques and whole-cell microscopy, this review will survey current advances in the methodologies that underpin flagellar data analysis and theoretical flagellar mechanics, highlighting the diverse opportunities for future research that are emerging as a result.

### 1.1. Flagellar Mechanics

#### 1.1.1. Classical Fluid Dynamics

**Figure 1:**
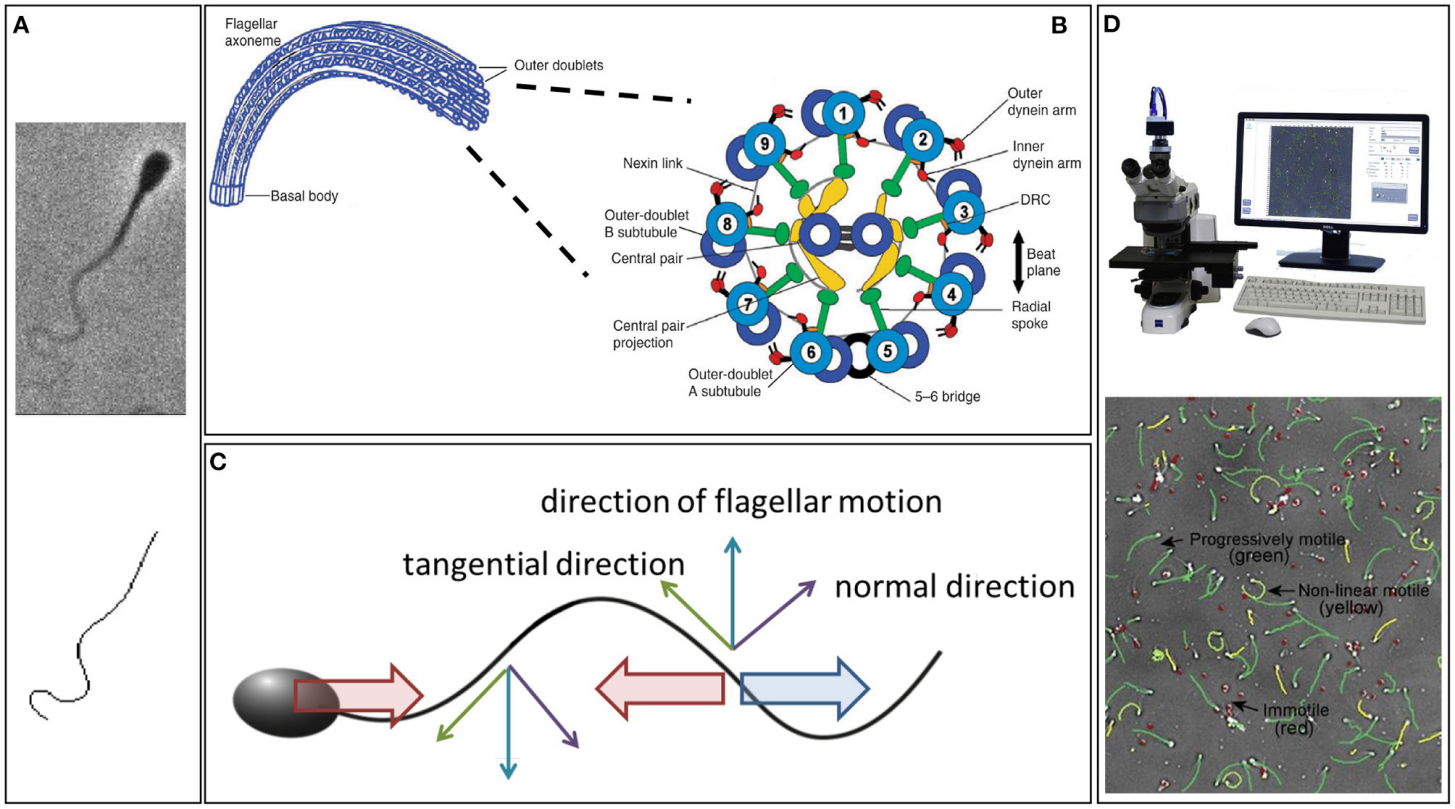
**(A)** An example of a phase contrast image of a swimming bull sperm (upper), with the digital capture of its flagellum (lower). **(B)** The internal structure of the flagellum, showing the dynein molecular motors. Their contraction induces a couple that acts to slide the microtubule doublets relative to each other, which, combined with the fact the microtubule doublets are constrained at the flagellum-cell body junction, induces flagellar bending (Summers and Gibbons, 1971; Brokaw, 1972a). **(C)** Resistive force theory, which simply relates local flagellum velocity, in the tangential and normal directions, to the forces exerted on the surrounding fluid. The thin upward blue arrow is the flagellum velocity, with the tangential and normal components represented by green and purple thin arrows, respectively. Noting that twice as much force per unit length is required to move the flagellum through the fluid perpendicular to itself compared to tangentially, the upwards-moving flagellum thus exerts a net force on the fluid in the horizontal direction to the right (large blue arrow); similarly for the thin downward blue arrow for the downwards flagellum velocity. Hence, an equal and opposite drag force acts leftwards on the flagellum (leftward red arrow). If the sperm was stationary this would violate Newton's second law, since the inertial term (mass × acceleration) is negligible, entailing that the total force on the cell must be zero. Thus, the sperm must move to the left at a speed that ensures that the additional drag from this motion (rightward red arrow) balances out the leftward force on the flagellum. Analogous reasoning can be used to find the sperm's vertical and angular velocities in terms of the flagellar beat pattern and, thus, the sperm trajectory can be constructed from knowledge of its beat pattern. **(D)** An example CASA system and cell tracking. **(A)** is republished from Walker et al. (2020b) with permission, under the terms of the Creative Commons Attribution License https://creativecommons.org/licenses/by/4.0/. **(B)** is republished with the permission of The Company of Biologists Ltd from Lindemann and Lesich (2010), permission conveyed through the Copyright Clearance Center, Inc. **(D)** is reprinted from Amann and Waberski (2014), with permission from Elsevier.

The swimming of sperm is characterised by physical scales where viscosity dominates inertia, the complete opposite to human swimming (Taylor, 1951). Hence, whilst everyday intuition does not apply to the microscale world of sperm swimming, the underlying fluid mechanics is in fact much simpler in this case. Indeed, at each instant in time, the fluid dynamics can be determined solely from the instantaneous velocity of the flagellum, independent of the history of the flagellar beat pattern. It is also *linear*, which in this context means that, *ceteris paribus*, doubling the frequency of flagellar beating will also double the swimming speed. The existence of such simple relations has enabled numerous rapid developments of Gray and Hancock (1955)'s seminal method, known as *resistive force theory* (Figure 1C), establishing quantitative links between the flagellar beat pattern and both the sperm's swimming speed and behaviour (for example, Rikmenspoel, 1965; Brokaw, 1972b; Suarez et al., 1991; Elgeti et al., 2010; Ishijima, 2011; Curtis et al., 2012). A key component of this theory is the notion of anisotropic drag, with approximately twice as much force being required to push the flagellum in its normal direction compared to its tangential direction (Gray and Hancock, 1955). This ultimately gives rise to the propulsion of a swimming spermatozoon (Figure 1) and can be observed by performing the simple experiment of moving a thin stick through syrup parallel and perpendicular to its length.

In particular, Gray and Hancock's resistive force theory framework assumes complete knowledge of the flagellar beat pattern, which is then used to predict the cell behaviour, rather than considering the fundamental question of how the beat pattern forms. Nonetheless, it provides a fundamental understanding of how beat patterns govern cell behaviour and also the energetics of motility, the latter by enabling the calculation of the mechanical energy and power required for sperm swimming (Ishimoto et al., 2018; Gallagher et al., 2019). It has a major advantage over other approaches due to its remarkable simplicity, though it accordingly makes numerous important assumptions. One pertinent example is that the surrounding medium is Newtonian in nature, such as water or a water-based physiological electrolyte, a common assumption more generally that we will later discuss in detail. A second and perhaps defining assumption, giving rise to the alternative name *local drag theory*, is that of locality, in that the viscous forces experienced at one point of the flagellum are taken to depend only on the velocity of the flagellum relative to the background fluid at that point. This, however, knowingly neglects the presence of, and any interactions with, not only the other parts of the flagellum but also the head of the sperm.

Hence, the level of accuracy in this resistive force theory is highly contingent on context, in that it should not be expected to retain accuracy for large cell bodies, highly curved flagella, nor multiple flagella, for example. Further, one should not expect much accuracy in its predictions of viscous drag close to the distal flagellum nor when the cell is swimming close to surfaces, since neither a finite length flagellum nor surface effects are included in the basic framework. The latter caveat is particularly of note for sperm microscopy, with imaging typically performed close to a coverslip owing to the fact that sperm are less likely to swim out of the focal plane in this setting, resultant of their well-known surface accumulation behaviours near flat boundaries. Despite this, high-accuracy resistive force theory for swimming bull sperm next to a plane has been reported (Friedrich et al., 2010), though with the notable caveat that a parameter within the theory was fitted to obtain the accuracy, whereas all parameters are theoretically fixed by physical principles in terms of the fluid viscosity and flagellum shape.

**Figure 2:**
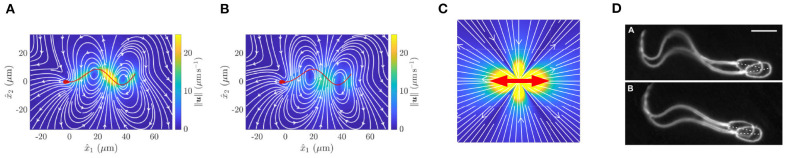
Fluid flow around a virtual spermatozoon, computed via **(A)** the boundary element method and **(B)** a coarse-grained model. **(C)** Flow induced by a point dipole model. The red arrows show the directions of force applied to the fluid, whilst color corresponds to flow velocity. **(D)** Pairwise swimming of bull spermatozoa. **(A,B)** reprinted from Walker et al. (2019b), Copyright (2019) by the American Physical Society. **(D)** republished with permission of the Company of Biologists Ltd from Woolley et al. (2009); permission conveyed through the Copyright Clearance Centre.

Whilst resistive force theory can, and has been, generalised to rigorously include surface effects (Brenner, 1962; Katz et al., 1975; Walker et al., 2019a), the resulting theory can be cumbersome to apply, losing the fundamental advantage of resistive force theory's simplicity that has allowed it to persist even to the present day. In particular, when relating flagellar beating to cell behaviour, there is a spectrum of classical methodologies to choose from that improve upon the limited accuracy and flexibility of resistive force theory, at least for Newtonian fluids (Gaffney et al., 2011). Furthermore, in contrast to resistive force theory, these improvements can also be used to determine the fluid flow induced by the sperm flagellum beating (Figure 2A). The most accurate and flexible is *computational fluid dynamics*, which is limited in accuracy only by computational resource, machine precision constraints, and the accuracy of the underlying physics, such as the common and broadly appropriate assumption of neglecting inertia on the grounds that it is subordinate to viscous effects and induces only tiny errors. The most common of such approaches for sperm motility are the *boundary element methods* (Pozrikidis, 2002), which have been extensively exploited to study sperm dynamics (for example Phanthien et al., 1987; Ramia et al., 1993; Ishimoto and Gaffney, 2014; Ishimoto et al., 2016; Walker et al., 2019b). However, such methodologies suffer from a relatively complex formulation, in terms of both the underlying mathematics and scientific computation.

An intermediate on the spectrum of accuracy, flexibility, and simplicity for methodologies is *slender-body theory*, which represents the flagellum via a curve of negligible cross-sectional radius, as developed by Keller and Rubinow (1976), Johnson (1980), and Tornberg and Shelley (2004), with numerous applications to sperm (Higdon, 1979; Johnson and Brokaw, 1979; Dresdner et al., 1980). Especially when coupled with the use of boundary element methods for the cell body (Smith et al., 2009a), this incurs only very small errors in predictions of the viscous drag, except at the distal end of the flagellum, and can readily accommodate a single flat surface. However, slender-body theory does not readily generalise to multiple swimmers, more complex confining geometries, or a non-trivial rheology of the surrounding fluid.

An interesting and useful generalisation of the boundary element method that has emerged in the past 20 years is the *regularised boundary integral method* (Cortez, 2001; Cortez et al., 2005), which differs from the original approach in a subtle way. In the original theory, in its most simple “single-layer” form, one can interpret the solution as the flows and forces induced by a collection of point forces in the fluid (Pozrikidis, 1992). True point forces present numerous challenges for computational algorithms, so the regularised boundary element method instead considers a collection of regularised forces, where the force is spread out in space, rather than acting at a single point, though this spread is highly localised. This leads to substantially simpler algorithms, generally at the cost of a limited but uncertain loss of accuracy, which depends on the fine details of how the force is spread out.

More generally, this framework has been extended to a regularised boundary integral method with singularities other than Stokeslets, with early examples developed by Ainley et al. (2008) and Smith (2009). There is also a further generalisation for sperm that we refer to as *regularised slender-body theory*, where the singularities are placed on the flagellum centreline. Compared to standard slender-body theory, this generalisation inherits ease of implementation at the expense of an uncertain loss of accuracy, and has also found substantial application in the modelling of sperm motility (Gillies et al., 2009; Smith et al., 2009a; Cortez and Nicholas, 2012).

#### 1.1.2. Elastohydrodynamics and Emergent Beat Patterns

As exemplified by Machin's study, there is more to the theory of sperm motility than simply how the flagellar beat pattern dictates the cell movement and the flow of fluid around the cell; there is the key question of how the flagellum beat pattern is generated. This is not independent of the surrounding fluid, with the viscous drag playing a fundamental role, as can be seen from differences in beat pattern between sperm surrounded by media of distinct viscosities (Suarez et al., 1991; Smith et al., 2009b). This dependence is further emphasised by the change in mechanical power output, which, for human sperm, increases by a factor of four on comparing swimming in a watery medium and a methylcellulose mucus analogue (Ishimoto et al., 2018), with the regulation of the dynein forces thereby also being modified by viscosity and, more generally, the resistance of the surrounding fluid. This is additionally supported by the observation that sperm flagella can synchronise when they are sufficiently close within media that present a high viscous and elastic resistance (Tung et al., 2017), demonstrating that the forces induced by the medium can effect the timing of the flagella beating.

Hence, the formation of the sperm beat pattern is multifacted, with its study requiring detailed consideration of the spermatazoan mechanical response to drag from the surrounding fluid, its own passive flagellar restoring forces, and the active forces and couples generated by its internal molecular motors. In turn, this entails that such a programme of research may be readily broken down into two distinct parts. The first is the fluid-flagellum interaction problem: given known active forces and torques, how does a flagellum beat and move in a viscous fluid environment where, as usual, fluid inertia is negligible? The second looks to query how to model the dynein molecular motors (Figure 1B), the forces and torques they exert, and their regulation, each of which may be coupled to the shape of the flagellum.

The first aspect of this programme concerns modelling the coupling of an elastic deforming filament to the flow of a viscous fluid. This is known as the *elastohydrodynamical problem*, predominantly considered in the inertia-free context appropriate for spermatozoa, which, here, is an excellent approximation. Most commonly, and up until relatively recently, the elastohydrodynamical problem has been pursued using the resistive force theory approximation (Rikmenspoel, 1971, 1978a; Brokaw, 1972b; Lindemann, 1994a; Fu et al., 2008, 2009; Gadêlha et al., 2010). Accordingly, this inherits all the inaccuracies, limitations, and lack of broad applicability described for resistive force theories, whilst moving beyond such local hydrodynamic theory has been, and broadly remains, a methodological challenge. One non-local approach has been the use of immersed boundary methods (Dillon et al., 2007), though these are very computationally demanding for elastic filaments, as opposed to two-dimensional sheets, whilst a further approach has been to use particle-based models with rod-and-spring representations of the flagellum (Elgeti et al., 2010). Tornberg and Shelley (2004) also demonstrated how slender-body techniques could be utilised for the elastohydrodynamic problem for filaments, though this has not been widely adopted in studying sperm motility. In contrast, a technique that has been adopted in this context takes advantage of regularised slender-body theory (Olson et al., 2013; Simons et al., 2014, 2015). However, once coupled with a sperm head and a confining surface, the inclusion of elastohydrodynamics within this methodology is observed to be very demanding in terms of computational resource (Ishimoto and Gaffney, 2018a). Seeking to overcome this computational hurdle, more-recent work has focussed on the development of algorithms that can solve elastohydrodynamical problems with much greater computational efficiency, as we shall touch upon later in Section 2.

The second component of the above programme—modelling molecular motor activity and its regulation—is a complex, multiphysics, multiscale problem that is subject to multiple competing theories and significant numbers of parameters that are not independently measured, as well as substantial inter-cell variation. There have been diverse studies by many authors that focus on how to represent the dynein dynamics, with examples including curvature control (Brokaw, 1975; Hines and Blum, 1978), multistate molecular motor models (Murase et al., 1989; Camalet and Jülicher, 2000; Dillon et al., 2007; Riedel-Kruse and Hilfinger, 2007), and Lindemann's geometric clutch hypothesis (Lindemann, 1994a,b, 2002), together with the latter's mathematical reincarnation by Bayly and Wilson (2014, 2015).

All such dynein models have been limited by the use resistive force theory, or the constraints of a 2D immersed boundary method in the case of Dillon et al. (2007). However, whilst such models are unquestionably hindered by the difficulties of the elastohydrodynamical problem, reviewing their scientific development is beyond the scope of this current review, but is lucidly detailed by Lindemann and Lesich (2010).

### 1.2. Computer-Assisted Sperm Analysis

Sperm microscopy presents difficulties in the high frequency of the flagellar beat pattern relative to the sensitivity of the human eye and the small diameter of the flagellum, which approaches the conventional resolution of light optics (Gray, 1955). To circumvent these challenges, early imaging modalities typically relied on darkfield microscopy and, before the emergence of sufficiently high frame rate cameras, stroboscopic illumination (Gray, 1955; Rikmenspoel et al., 1960; Sleigh, 1962). This technique was refined by Rikmenspoel to achieve cinemicroscopy with flagella imaged at 200 frames per second by the mid 1960s (Rikmenspoel, 1965), progressing to 400 frames per second Rikmenspoel (1978b) as well as being adopted by Sleigh, amongst others, for studies of both cilia and flagella (Sleigh, 1974; Sanderson and Sleigh, 1981). Another popular approach emerged with the Nobel prize winning revolution of phase-contrast microscopy, which was developed in the 1930 and 1940s (Zernike, 1955). It enables remarkable spatial resolution at the cellular and sub-cellular scale and has been widely exploited for the imaging of the slender flagella of sperm cells (for example Katz et al., 1978; Overstreet et al., 1979; Figure 1A, upper).

However, the conversion between imaging capability on the one hand and meaningful summary statistics of sperm motility on the other required labour-intensive manual analysis of time exposure photomicrographs (Overstreet et al., 1979) or frame-by-frame by-hand analysis of cine films (Rikmenspoel, 1965, 1978b; Katz et al., 1978), as reviewed by Amann and Katz (2004). The laborious nature of studying sperm with such methods was alleviated with the emergence of *Computer-Assisted Sperm Analysis (CASA)* and *Computer-Assisted Sperm Motility Analysis (CASA-Mot)*, Figure 1D, in the 1980s, as summarised by Davis and Katz (1989). The technology finds extensive application in theriogenology and andrology, as well as reproductive toxicology and semen marketing for livestock breeding (Mortimer, 1997; Mortimer et al., 1998; Amann and Waberski, 2014), though its use in clinical diagnostics is far from fully accepted (Gallagher et al., 2018).

Whilst CASA-Mot generates numerous and standardised measures of sperm swimming, such as speeds, yaw, and trajectory curvature (WHO, 2010), it almost exclusively focuses on the spermatozoon head and its trajectory. However, with the flagellum and its waveform being fundamental for sperm motility, the beat pattern has been the subject of extensive scientific enquiry since its discovery, though the details of even the shapes formed during its complex and varied beating patterns remain elusive. Indeed, since the advent of appropriate microscopy techniques, the standard approach to quantifying the shapes of beating flagella has been simple: trace out, by hand, the visible flagellum in each captured frame of microscopy data (Ishijima et al., 2002; Vernon and Woolley, 2002, 2004). Understandably, the significant time and human investment required to gain even moderate quantities of digitised beating data in this way limited the scope and power of kinematic analysis. Recent developments, which we later summarise, have naturally striven to overcome this, with a sample automated digitisation shown in Figure 1A.

Such modern methods promise to greatly increase the quantity and fidelity of flagellar data available to the community. Realising the full potential of this data, however, will itself necessitate significant complimentary developments in the mathematical modelling of motility and related theories. This need spans many aspects of sperm motility analysis, from the removal of the restriction to Newtonian fluid media to overcoming the drawbacks of standard elastohydrodynamical algorithms. Hence, in what follows, we will survey both a number of recent efforts to address some of these challenges and the opportunities for further and future development that these methodological advances unveil.

## 2. The Evolving Methodological Landscape

### 2.1. Population-Level Modelling

#### 2.1.1. Interacting Swimmers

It has long been known that sperm cells swim together, as exemplified by the microscopy of Woolley et al. (2009), reproduced in Figure 2D. In the middle of the last century, the importance of hydrodynamic interactions between cells was suggested by the now classical theoretical analysis of Taylor (1951). Since then, innumerable studies have sought to explore the role of hydrodynamics in the collective swimming of spermatozoa, but the fine details of these interactions remain uncovered. Indeed, the pairwise swimming of even two individuals is not fully elucidated, a necessary precursor to the accurate population-level modelling of swimming sperm. Nevertheless, multiple approaches have been utilised to investigate, with associated levels of approximation, the motion of collections of spermatozoa.

The coarsest model of a single swimmer is the so-called *point-dipole* model (Lauga, 2020), which, in essence, simplifies the cell down to a single point, with the surrounding flow being modelled by two opposing forces, as shown in Figure 2C. This representation effectively averages the more complex time-varying flow around a spermatozoon, shown in Figure 2A, in doing so almost entirely neglecting the morphology of the swimming cell. These models inherently afford great simplicity and scalability, whilst providing an accurate picture of the hydrodynamics of a swimmer when viewed from the far field, the combination of which has resulted in their widespread use. Despite their apparent crudeness, their utility has been repeatedly highlighted, not least by the ability of these simple representations to explain the attractive hydrodynamic interactions between cells that swim in parallel to one another (Lauga and Powers, 2009), though this does not guarantee the emergence of experimentally noted pairwise swimming.

Arguably representing the opposite end of the modelling spectrum, the computationally intensive *boundary element methods* enable the complex spermatozoan shape to be represented numerically (Pozrikidis, 2002), giving rise to accurate quantifications of the fluid flow around spermatozoa and the accompanying hydrodynamic interactions. For example, this methodology was used to produce the intricate instantaneous flow field displayed in Figure 2A, significantly distinct in character from the time-averaged flow field of Figure 2C. This accurate approach was recently used to simulate the motion of two identical model sperm with planar flagellar waveforms (Walker et al., 2019b), which concluded that two cells swimming side-by-side are attracted towards eventual collision, whilst those that are above and below one another (with respect to the plane of the flagellar beat) can swim stably at a certain distance apart. This simulation study highlights the subtle and intricate details of the hydrodynamic interactions between swimming sperm, though such a high fidelity simulation cannot be reasonably extended to population-level analyses due to the overwhelming numerical cost.

An intermediate approach, seeking to balance accuracy and efficiency, involves coarse graining the flow around a spermatozoon, representing the dynamics of the fluid by a small number of simple flow constituents (Ishimoto et al., 2017). The details of this method are somewhat involved, though their success in improving accuracy over the minimal point-dipole is easily evidenced, as can be seen by comparing the flow fields of the point-dipole (Figure 2C) and the coarse-grained approach (Figure 2B). This methodology can be applied to sperm in multiplicity (Ishimoto et al., 2018), enabling theoretical studies of small populations of spermatozoa with heightened accuracy and efficiency when compared with point-dipole and boundary element methods, respectively.

A subtlety in the common use of each these methods, however, is that they often rely on knowledge of the flagellar beat. The extension of swimmer-swimmer interactions to an elastohydrodynamic setting, where the shape of the flagellum is influenced by its elastic properties as well as interactions with the surrounding fluid, remains challenging. However, the exploration of this coupling, has, and continues to be, the subject of much active research, from early models of *swimming sheets* (Fauci and McDonald, 1995) to more recent theoretical analyses (Elfring and Lauga, 2009, 2011), the latter works highlighting the enhancement of flagellar synchronisation by elasticity. Elastohydrodynamic modelling has since been extended to consider planar motions of flagella (Llopis et al., 2013; Goldstein et al., 2016; Taketoshi et al., 2020), with Taketoshi et al. (2020) extending the boundary element method to include flagellar elasticity. This latter work again explored pairwise dynamics, numerically concluding that spermatozoa enjoy increased swimming speed when beating in synchrony, in contrast to the similar but conditional results of the prescribed-beat study of Walker et al. (2019b). Further, three-dimensional studies have also been initiated (Simons et al., 2015), though there remains significant scope for the investigation of the effects of rheology, confinement, multiplicity, and the details of the driving force behind the spermatozoan beat.

#### 2.1.2. Collective Behaviours

**Figure 3:**
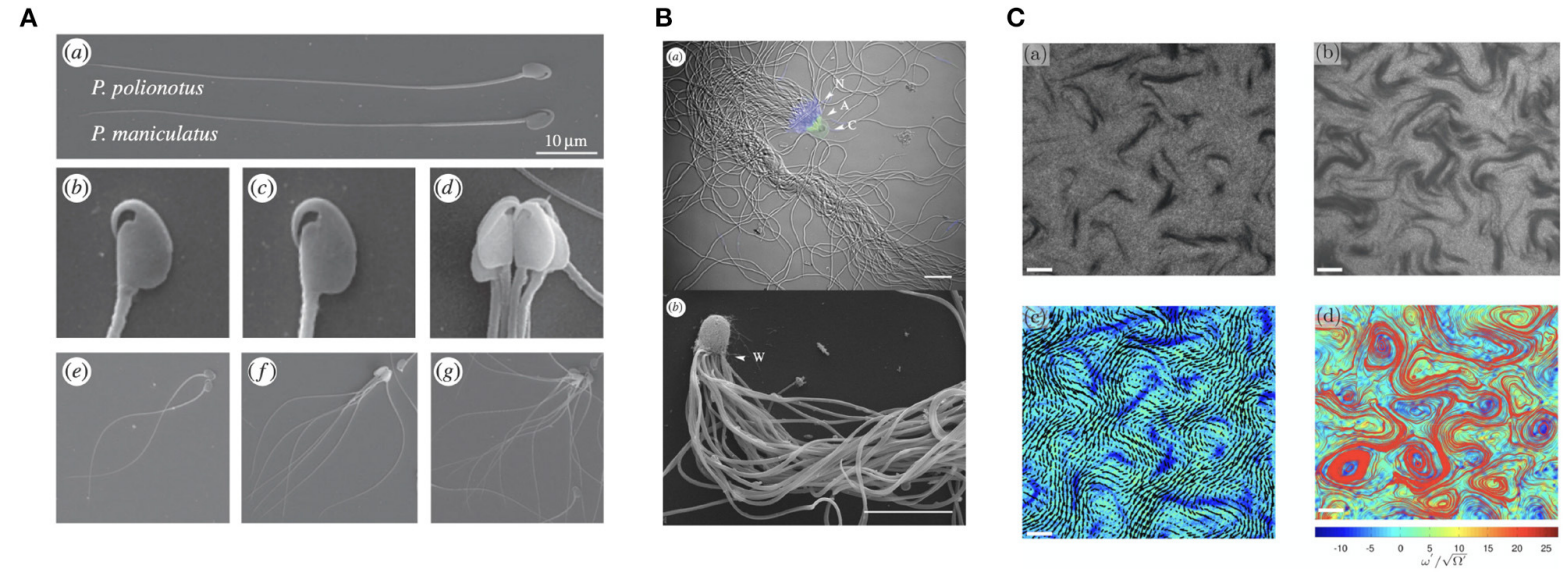
Varieties of sperm collective behaviors. **(A)** A multiswimmer sperm train and the hook-like morphology of some murine spermatozoa. **(B)** Crowded sperm bundles of potentially hundreds of individual swimmers. **(C)** The emergence of sperm turbulence, found in dense suspensions. **(A)** is republished from Fisher et al. (2014), with permission under the terms of the Creative Commons Attribution License http://creativecommons.org/licenses/by/3.0/. **(B)** is republished with the permission of The Royal Society (U.K.) from Pearcy et al. (2014); permission conveyed through the Copyright Clearance Center, Inc. **(C)** is reprinted from Creppy et al. (2015), Copyright (2019) by the American Physical Society.

With spermatozoa often present in vast numbers (Zinaman et al., 2000), it is unsurprising that a wide variety of collective behaviours have been observed. Shown in Figure 3A, a particularly remarkable example is the phenomenon of *sperm trains*, collections of up to around ten cells that adhere to one another at the head, which occur in some species of rodent (Moore and Taggart, 1995; Moore et al., 2002). These are thought to form due to the hook-like morphology of the spermatozoon head in these organisms, whilst pairwise adhered swimming has been reported to confer increased swimming speed over lone cells in *Monodelphis domestica*, upwards of 20%. This is in agreement with the elastohydrodynamic simulations of Cripe et al. (2016), which considered the motion of two flagella adhered at the base, though these results are significantly dependent on parameters such as interflagellar distance and the angle between the adhered flagella.

Further, in some monotreme, ants, and other species, it has been observed that spermatozoa can form a large bundle-like structure, as showcased in Figure 3B, containing more than 100 cells (Djakiew and Jones, 1983; Burnett and Heinze, 2014; Pearcy et al., 2014). These *sperm bundles*, as well as sperm trains, have also been studied from the point of view of elastohydrodynamics (Yang et al., 2008, 2010; Schoeller et al., 2020), with head-head adhesive interactions also having been modelled by Fisher et al. (2014), the latter being noted to be of significance for the competitive viability of such entrained collections of spermatozoa. An additional modelling study suggests the importance of spermatozoan head geometry in the stability and motility of these aggregates (Pearce et al., 2018), with hydrodynamic interactions more generally being highly dependent on cellular morphology. It is important to note, however, that the adhesion that gives rise to these clusters does not appear necessary for their formation in general, with Tung et al. (2017) having reported the aggregation of spermatozoa in the absence of clear adhesion; rather, the observed groupings were transient, with sperm transitioning between clusters over time.

Other collective phenomenon include *sperm vortices*, swirling structures that can arise due to asymmetric flagellar beating of certain species near a substrate (Riedel et al., 2005; Yang et al., 2014b), and *sperm turbulence*, which can be found in dense suspensions both experimentally and theoretically (Creppy et al., 2015, 2016; Schoeller and Keaveny, 2018). This structured turbulence, pictured in Figure 3C, appears reminiscent of well known bacterial turbulence (Wensink et al., 2012) and the collective dynamics of active rods (Saintillan and Shelley, 2007), though the statistical features of these phenomena differ slightly.

### 2.2. The Sperm Microenvironment

Further to the presence of nearby swimmers, more-fundamental aspects of the spermatozoan microenvironment can impact significantly on the motion and behaviours of the swimming cell. For instance, the chemical components of the surrounding environment also can affect the motility of sperm, in particular inducing turning and chemotactic guidance, as first demonstrated by Miller (1966) in hydroids. Studies of the associated flagellar kinematics followed relatively shortly, with the demonstration that chemotactic turning is concomitant with asymmetric flagellar beat patterns (Miller and Brokaw, 1970), again in hydroids. The functional significance of chemotaxis in terms of animal sperm guidance to the egg was first reported in Ward et al. (1985)'s study of sea urchin, whilst the first direct support of the underlying mechanism, in terms of the modulation of intra-cellular calcium, was presented by Cook et al. (1994). The time taken to begin to directly evidence the underlying mechanism of sperm chemotaxis via its impact on intracellular calcium emphasises the challenge in elucidating the systems biology of sperm guidance (Kaupp et al., 2003), as does the observation that this is still not fully resolved to date (Priego-Espinosa et al., 2020).

Nonetheless, it is fully recognised that chemotaxis is a key mechanism of sperm guidance (Eisenbach and Giojalas, 2006; Friedrich and Jülicher, 2007; Cosson, 2015; Jikeli et al., 2015; Hussain et al., 2016; Kaupp and Alvarez, 2016) that is of particular relevance for external fertilisers, where chemoattractants are released from the ovum, in turn inducing a modulation of the sperm flagellar waveform that promotes guidance towards an egg (Shiba et al., 2008). These chemoattractants can be advected by the flow induced by the flagellum and the background fluid, with the latter inducing a spread over a wide region, potentially enabling long range signalling (Riffell and Zimmer, 2007; Zimmer and Riffell, 2011). For instance, fluid shear in marine environments can induce a filamentous region with a strong concentration of molecules extending from the egg (Bell and Crimaldi, 2015). In turn, this can promote sperm cell chemotaxis at larger distances from the egg, with theoretical modelling showing that the moderate shear rate of coastal waters is optimal for sperm-egg encounter rate (Lange and Friedrich, 2021). Nonetheless, the study of the impact of chemoattractants and their transport on sperm behaviour presents numerous significant challenges that are yet to be wholly addressed, including even the measurement of chemoattractants *in vivo*.

Perhaps more fundamentally, the microenvironment can impose mechanical constraints on swimming. In the remainder of this section, we will consider two such factors: the geometrical confinement experienced by the swimmer, such as in a microdevice or the female reproductive tract, and the properties of the fluid media in which it swims, with different media giving rise to greatly distinct beating gaits.

#### 2.2.1. Confinement

A typical, but not ubiquitous, limitation of optical microscopy is that data is acquired in a single focal plane. As free-swimming spermatozoa need not move in this same plane, the acquisition of swimming data can be challenging. This has led to sperm being imaged in confined environments, such as near a substrate or coverslip, which serves to limit the swimmer motion out of the focal plane, enabling swimmer behaviour to be captured in high fidelity. This arises due to the tendency of sperm to swim near a boundary, well-known to occur for glass substrates for over half a century (Rothschild, 1963). Simple theoretical arguments, using the aforementioned point-particle models, predict this behaviour, with hydrodynamic interactions drawing the swimmer close to the boundary Lauga (2020). However, these models fail to capture the fine but significant details of the hydrodynamic interactions between the boundary and a spermatozoon, with the details of the flagellar waveform known to have a crucial impact on the realisation of accumulation behaviours via hydrodynamics alone (Smith et al., 2009a; Ishimoto and Gaffney, 2014).

In addition to the potentially subtle effects of hydrodynamics, there are further mechanical interactions between boundaries and swimmers. Example such mechanisms are contact and adhesive forces, which, much like in the case of swimmer-swimmer interactions, have not been well explored in theoretical studies, though numerical works have documented the interplay between adhesion and hyperactivated beating patterns in realising detachment from a surface (Curtis et al., 2012; Simons et al., 2014; Ishimoto and Gaffney, 2016).

A further, often experimentally undesirable impact of boundary proximity is modification of the flagellar beat. A drastic change in beating was reported by Woolley (2003), with a three-dimensional helical motion of the flagellum being suppressed to a two-dimensional planar gait due to interactions of the spermatozoon with the boundary. This tendency of boundaries serving to reduce non-planar components of beating has also be affirmed by more recent observations (Su et al., 2012; Bukatin et al., 2015; Nosrati et al., 2015). From a theoretical perspective, elastohydrodynamic studies have sought to investigate this phenomenon (Fauci and McDonald, 1995; Elgeti et al., 2010; Huang et al., 2018; Ishimoto and Gaffney, 2018a), concluding that the flagellar waveform can be modified by hydrodynamic interactions with boundaries, though further experimental investigation is required in order to clarify the effects of boundaries on the flagellar gait.

In applications, and certainly *in vivo*, the geometry of confinement need not be as simple as a plane wall. In microdevices, sperm often experience interactions with sharp corners, which give rise to both scattering and accumulation behaviours (Kantsler et al., 2013; Nosrati et al., 2016; Bukatin et al., 2020). Elastohydrodynamic simulations of swimmers near such corners have recently been conducted (Montenegro-Johnson et al., 2015; Rode et al., 2019), though with assumed two-dimensional flagellar waveforms. Experimentally, more complex geometry has been examined, for example in sperm sorters (Denissenko et al., 2012; Tung et al., 2014; Kamal and Keaveny, 2018), though this complexity has inhibited numerical exploration of the same intricate environments. For instance, unexplored theoretically to the best of our knowledge, remarkable *in vivo* experiments of Yang and Lu (2011) exemplify the drastic effects that severe confinement can have on sperm motility in Drosophila, whose long sperm are able to move rapidly in the contorted female reproductive tract whilst being practically immobile in free artificial media (Pitnick et al., 1995; Lu, 2013).

#### 2.2.2. Rheology

A key influence on the waveform exhibited by a spermatozoon is the viscosity of the surrounding fluid, as the balance between elastic and viscous forces changes. A prominent effect is on the amplitude of the flagellar waveform, with high viscosity leading to flagella that appear to bend more readily, which has also been seen in theoretical studies (Fu et al., 2007; Gadêlha et al., 2010). A similar effect occurs as the result of reduced stiffness towards the distal end of the flagellum (Gadêlha and Gaffney, 2019).

**Figure 4:**
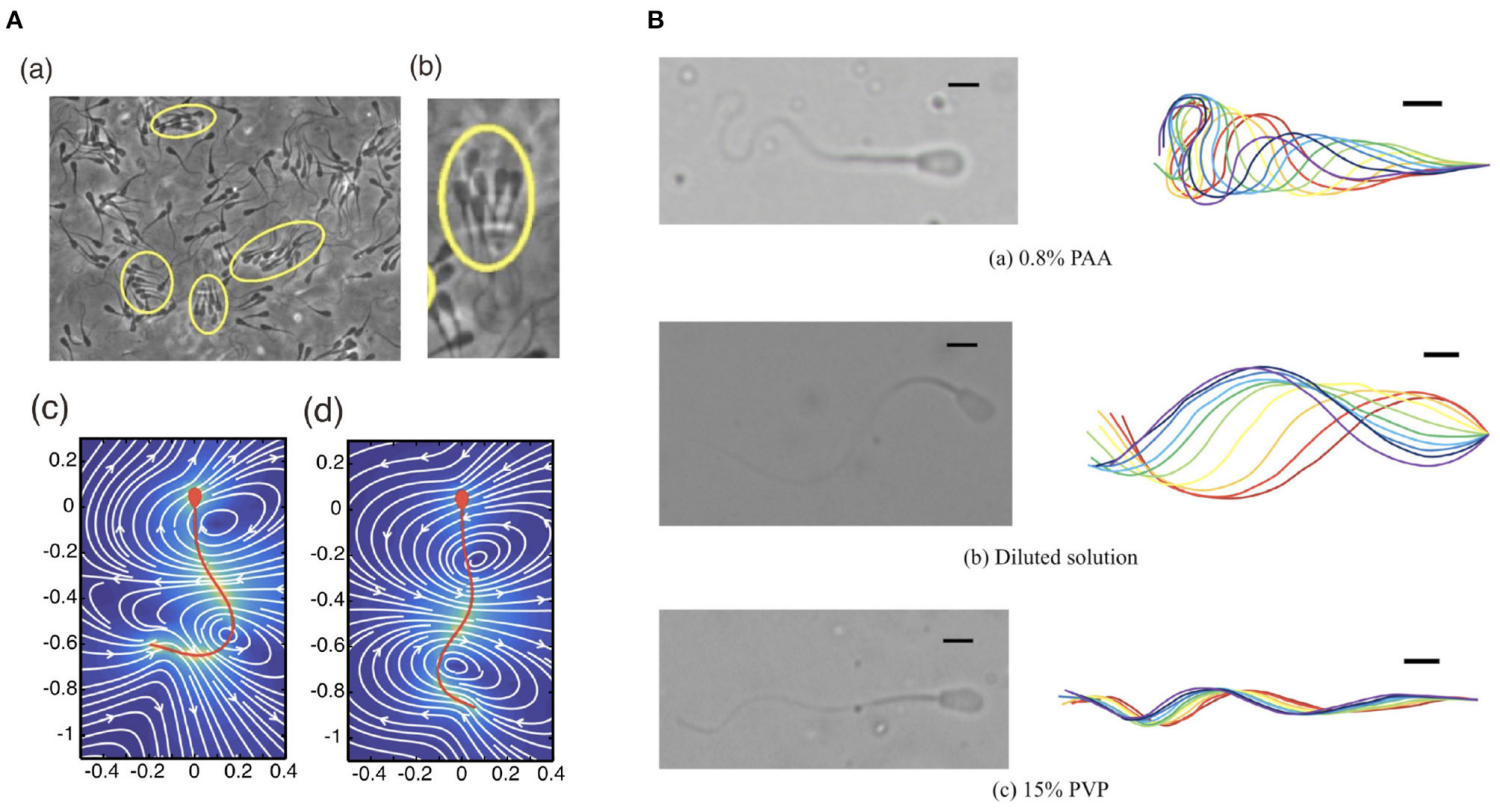
Different waveforms in different environments. **(A)** (a,b) Bull sperm cluster in a viscoelastic medium (Tung et al., 2017) and the flow of (c) low and (d) high viscous medium around human sperm cells (Ishimoto and Gaffney, 2018b). **(B)** Different waveforms seen in bull sperm cells in different rheological media (Hyakutake et al., 2019) **(A)** (a,b) republished from the works of Tung et al. (2017) and **(A)** (c,d) republished from the works of Ishimoto and Gaffney (2018b), all with permission under the terms of the Creative Commons Attribution License http://creativecommons.org/licenses/by/4.0/. **(B)** reprinted from Hyakutake et al. (2019) with permission from Elsevier.

Additionally, swimmer dynamics may also be drastically altered by more complex fluid rheology (Smith et al., 2009b; Hyakutake et al., 2019), such as the potentially elastic nature of a fluid due to solvent molecules, broadly termed *viscoelasticity*, in contrast to usual watery medium, termed a Newtonian fluid, as discussed in the previous sections. A complex fluid that cannot be described simply by the Newtonian model is called a non-Newtonian fluid. One of the simplest models for a non-Newtonian medium is a linear Maxwell fluid, which contains a single parameter that encodes a relaxation time due to the elastic property of the medium. In the linear Maxwell fluid model, the rheology of the fluid does not directly influence the hydrodynamic interactions (Fulford et al., 1998; Ishimoto et al., 2017), though does impact on the forces felt by a swimming cell (Ishimoto and Gaffney, 2016). More generally, swimming in non-Newtonian media can result in non-trivial changes to the speed of progression and the emergent waveforms, exemplified in Figure 4. In turn, these potentially result in large departures from normal Newtonian behaviour, both in individual and collective dynamics (Li and Ardekani, 2016; Thomases and Guy, 2017; Ishimoto and Gaffney, 2018b; Ishimoto et al., 2018), with a particular example being the enhancement of coherent multiswimmer structures in a non-Newtonian fluid (Tung et al., 2017).

Ultimately, the investigation of swimming in complex fluids requires significant advances in modelling methods and numerical schemes, with non-Newtonian fluids being generally more difficult to study. This affects not only simulation at scale, necessary for investigating collective behaviours, but also the study of individual swimmers, which remains challenging.

### 2.3. Computer-Assisted Beat-Pattern Analysis

#### 2.3.1. Digitising the Flagellar Beat

As summarised in section 1, the task of studying the flagellar beat has classically been laborious, requiring vast amounts of researcher time to trace flagellar shapes from microscopy. To overcome this barrier to large-scale quantitative analysis, a host of computer-assisted methods have been developed, reducing or removing the need for researcher interaction with the raw dataset. One early approach utilised a television camera and video digitiser for the processing of rephotographed microscopy images, with manual intervention for dust spots and film scratches (Rikmenspoel and Isles, 1985). A further early approach was that of Baba and Mogami (1985), which used pixel intensity measurements to trace out a flagellum from an initially selected basal point. Both approaches were a significant improvement on previous manual techniques and allowed sufficient accuracy for the quantification of flagellar curvature. In particular, the associated software developed from Baba and Mogami (1985)'s study, BohBohsoft, has persisted and enabled numerous studies requiring flagellar digitisation, such as Wood et al. (2005) and Shiba et al. (2008).

More recently, a wide range of refined semi-automatic schemes, requiring further-reduced user input, and even fully automatic unsupervised methods, have become available for tracing out a slender flagellum-like object from videomicroscopy. A selection of these approaches are tailored to the morphology and characteristics of spermatozoa (Smith et al., 2009a; Yang et al., 2014a; Hansen et al., 2018; Gallagher et al., 2019), whilst others are somewhat more general (Hongsheng et al., 2009; Goldstein et al., 2010; Xu et al., 2014; Xiao et al., 2016; Walker et al., 2019c); an example output of one of the latter techniques is reproduced in Figure 1A. The development of these software tools and approaches, in combination with improvements in the fidelity of videomicroscopy, has newly enabled studies at scale of the details of flagellar beating in a variety of organisms, including bovine and human spermatozoa (Gallagher et al., 2019; Walker et al., 2019d, 2020b), each analysing hundreds of individual swimmers, with the potential for significant future application and extension.

#### 2.3.2. Summary Statistics

This marked increase in the availability of flagellar beating data, which does not form part of a traditional CASA or CASA-Mot frameworks, motivates the development of a new generation of semen analysis techniques, as noted in the thorough review of Gallagher et al. (2018). Recent methods have sought to incorporate simple summary measures of the flagellar beat, augmenting traditional CASA and CASA-Mot statistics with quantities such as wavelength and beat frequency (Gallagher et al., 2019; Walker et al., 2019d). However, whilst the simplicity of these descriptors is attractive and they can be readily computed, they each implicitly assume particular characteristics of the flagellar beat, which, as we will see, need not hold in practice.

The first and perhaps most subtle assumption is well illustrated by the notion of wavelength, and concerns the problem of definition. To illustrate this, consider the waving motion of a simple travelling sine wave, familiarly written as *y* = sin(*kx*−*t*) for position *x* and time *t*. This has a characteristic and well defined wavelength, here given via the quantity *k*. Whilst similar such sinusoidal patterns have been classically used to caricature the flagellar beat, the true beating motion of a sperm cell is readily observed to not be quite so simple, as can be seen in Figure 4B. Further focussing on the upper panel of Figure 4B, it is clear that the concept of wavelength lacks unambiguous meaning in this context, with there being no familiar repeating shape as we move along the flagellum, as would be the case for a simple sine wave. As such, measurements, interpretation, and related discussions of wavelength should be treated with great care, with one study's notion of wavelength not necessarily synonymous with that defined in another analysis. Analogously, this carries over to similarly derived flagellum-wide quantities, such as beat amplitude.

The second commonplace assumption is somewhat more intuitive, and highlights a relatively unexplored aspect of flagellar beating: the evolution of beating over time. For example, though we might report the frequency of the flagellar beat as a single value, the actual frequency may be evolving with time. Of particular note, the recent study of Achikanu et al. (2019) tracked sperm motion over a considerable time period, identifying sustained behavioural switching that is more significant than we have hinted at here, highlighting the significance of careful temporal considerations of the spermatozoan beat and its descriptors.

#### 2.3.3. Whole-Flagellum Analysis

Whilst even refined and well defined summary statistics provide a readily digestible characterisation of a flagellar waveform, they necessarily omit much of the detailed beat pattern data available. Common techniques such as principal component analysis (PCA) can encode such complex waveform information in a number of coefficients and so-called modes, though at the expense of easy interpretation and simple methods of comparison between swimmers.

Two recent works, however, have sought to address the second of these two drawbacks, applying PCA not just to individual swimmers, but to whole cohorts of swimming cells in order to capture population-level beating information. One, that of Walker et al. (2020b), computed time-dependent PCA coefficients and applied standard hypothesis testing techniques to these quantitative measures, enabling statistical comparison between samples of bovine spermatozoa via the details of their beating, coining the term *computer-assisted beat-pattern analysis* (CABA).

Complementary to this, Guasto et al. (2020) utilised the PCA modes derived for the sperm of different species, from marine invertebrates to human, to compare the shape of the spermatozoan beat, which led to the suggestion of the importance of selective environmental pressure on shaping spermatozoan motility. With these methodological developments being so recent, there remains significant scope for the broader application of data-rich quantitative approaches to spermatozoan motility, from the statistical comparison of samples to querying flagellar form and function via computer-assisted beat-pattern analysis.

### 2.4. Elastohydrodynamic Advances

The computational simulation of flagellar elasticity and hydrodynamics is well-known to be a prohibitively difficult task (du Roure et al., 2019), requiring hours on large computing resources to perform even a single simulation (Ishimoto and Gaffney, 2018a). Recently, in an attempt to remove this obstacle to elastohydrodynamics, a new coarse-grained approach was proposed by Moreau et al. (2018). Indeed, this method successfully reduced computation times in 2D simulations down to seconds on laptop computers, improving efficiency by multiple orders of magnitude. The key advance of this framework was to computationally represent the elastic flagellum as a series of connected straight segments and then sum up the drag forces and elastic moments on each straight piece. This led to a simple and flexible set-up that could be rapidly simulated and readily extended to a variety of contexts.

Such was the increase in simulation speed and utility of the approach, this methodology has already been extended by multiple groups to include improved hydrodynamics (Hall-McNair et al., 2019; Walker et al., 2019a) and utilised for exploratory study (Neal et al., 2020). In the latter, tailored to spermatozoa, Neal et al. (2020) leveraged the computational efficiency afforded by this methodological advance to explore the effects of multiple parameters on the swimming efficiency of spermatozoa, concluding in particular that an inactive flagellar endpiece can increase the efficiency of swimming. Recent work has also lifted this approach into 3D (Walker et al., 2020a), which again realised orders of magnitude improvements in computational speed over contemporary 3D methodologies (Olson et al., 2013; Ishimoto and Gaffney, 2018a; Carichino and Olson, 2019). The full extent of these advances is yet to be realised, with the potential to greatly expand the scope of both theoretical and data-driven research into the dynamics of the flagellum.

## 3. Next-Generation Investigation

### 3.1. Refining Mathematical Models

#### 3.1.1. Subcellular Investigation

Advances in capability have naturally afforded advances in scope. For instance, the vast majority of existing theoretical research into spermatozoan motility incorporates known flagellar waveforms into computational models, deducing quantities such a swimming speed or efficiency. However, with elastohydrodynamic methods becoming more popular and, as noted, significantly more efficient, there is novel opportunity to move past kinematics, considering instead the molecular motor dynamics internal to the flagellum. In particular, the prospect now exists for much more extensive exploratory computational studies of this subcellular process, for which many hypotheses exists but none have been universally validated or agreed upon (Hines and Blum, 1978; Lindemann, 1994a; Riedel-Kruse and Hilfinger, 2007). There is also broad scope for the further investigation of the impacts of calcium dynamics on flagellar waveforms, having been recently considered theoretically (Olson, 2013; Carichino and Olson, 2019) and suggested to significantly modulate, or even disable, the flagellar beat (Corkidi et al., 2017; Sanchez-Cardenas et al., 2018).

However, of particular pertinence to regulatory models but pervasive more generally, the biologically realistic parameterisation of flagellar models represents an ongoing challenge for the community, with key measurements lacking for many of the relevant mechanical parameters. Indeed, whilst efforts have identified some material properties of flagella, such as bending and shearing resistance in some organisms (Minoura et al., 1999; Pelle et al., 2009), measurements of many quantities, such as torsional resistance, lateral compressibility, and lateral extensibility, are absent. Due to this lack of appropriate data, theoretical studies are commonly limited to simply estimating material parameters, such as is the case in the aforementioned work of Bayly and Wilson (2014), to Table 4 of which we direct the reader in order to illustrate the scale and scope of the absence of known material quantities, even in the context of Bayly and Wilson's idealised flagellar model. Hence, the detailed measurement of material properties of flagella, guided by and addressing the pressing needs of the theoretical community, represents a pertinent goal for future experimental investigation. Complimentary to this, and likely best realised via the strengthening of collaborative links between experimental and theoretical disciplines, additional efforts are warranted to make appropriate use of available measurements in mathematical models, with studies having often neglected realistic parameterisation, and therefore biologically relevant enquiry, in favour of more abstract exploration.

#### 3.1.2. External Influences

In addition to the detailed consideration of internal factors, future theoretical studies may realise high-fidelity coupling of external influences to the flagellar beat, such as fluid flows, which are the root of rheotaxis in spermatozoa, and chemoattractant-induced taxis (Miki and Clapham, 2013; Kantsler et al., 2014; Ishimoto and Gaffney, 2015; Hussain et al., 2016). For example, such investigations provide the opportunity to couple the aforementioned improvements in computational elastohydrodynamics and our understanding of molecular motor regulation to study the emergence of asymmetric beat patterns and sperm turning due to guidance cues (Alvarez et al., 2014; Bukatin et al., 2015), including the potential role of flagellar buckling (Gadêlha et al., 2010; Bukatin et al., 2015; Ishimoto and Gaffney, 2018a; Kumar et al., 2019).

Other aspects of the spermatozoan microenvironment also present notable challenges to the mathematical modelling community. For instance, theoretical study that accurately reflects the complex rheology of the female reproductive tract, which is potentially non-Newtonian and displays elastic properties, remains a particularly significant and unresolved challenge for the modelling community, with much contemporary research instead being of more direct relevance to tightly controlled *in vitro* systems. Any advances in this area would also be of much wider pertinence, with rheology being a major confounding factor in the study of cilia and flagella in many contexts, such as those involved in mucociliary clearance and development.

A further complicating factor of *in vivo* systems is their geometry and form, which present a theoretical and computational barrier to mathematical analysis. Whilst exact methodologies are available for studying the fluid flow in the simplest geometries, such as half-spaces or basic channels, similar such tools do not exist for the intricate morphologies found in biology. Whilst recent methods for numerical simulation seek to overcome this problem, such as the mesh-free approach of Gallagher and Smith (2018), accurate and detailed consideration of the shape of pertinent fluid environments remains lacking, requiring marked advances in the field of fluid mechanics as well as coupling to high-resolution imaging and rheological measurements.

### 3.2. Flagellar Analysis in 3D

**Figure 5:**
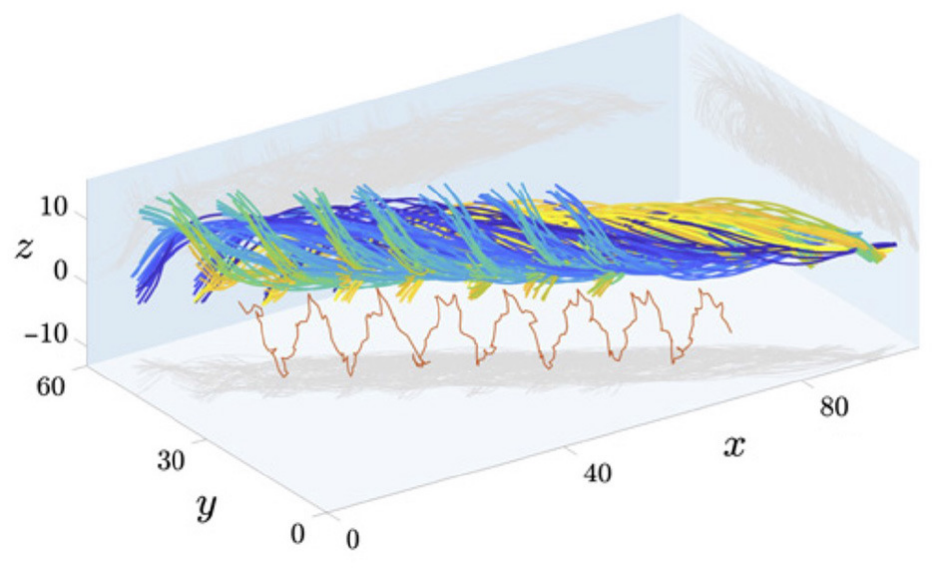
Captured data of the 3D beating of human spermatozoa near a coverslip, with different timepoints shown in different colours. Axes have units of micrometres and a projection of the mid-flagellar point is shown in red. Figure reproduced from the work of Gadêlha and Gaffney (2019), Gadêlha et al. (2020); Shilatifard and Yeagle (2020), with permission under the terms of the Creative Commons Attribution License http://creativecommons.org/licenses/by/4.0/.

Much of the flagellar analysis from videomicroscopy that we have described has been relevant to the most common output of such videomicroscopy: two-dimensional images. However, with the beating of spermatozoa not always being planar, a natural extension of these approaches is to three-dimensional flagellar motion. Indeed, three-dimensional beating data of high quality is becoming increasingly available in general (Su et al., 2013; Wilson et al., 2013; Silva-Villalobos et al., 2014; Hernandez-Herrera et al., 2018; Walker and Wheeler, 2019; Hansen et al., 2020), with holographic imaging having recently been used to study spermatozoa (Daloglu and Ozcan, 2017; Daloglu et al., 2018; Muschol et al., 2018). We showcase sample 3D imaging from Gadêlha et al. (2020) in Figure 5, though we note that the work of Gadêlha et al. (2020) has recently been retracted for reasons unrelated to the imaging reproduced here (Shilatifard and Yeagle, 2020; Gadêlha et al., 2021). With this increase in the availability of high-fidelity data, which we only expect to further develop in the coming years, the complimentary approaches of CASA and CABA can be readily extended to motion in three dimensions, broadening the scope of quantitative sperm analysis. This promises to enable new insights via kinematic evaluation of the flagellar beat in unrivalled detail, along with providing a means for further intersample and interspecies comparison that is applicable to general, non-planar motion. That being said, whilst the extension to three dimensions may represent the next generation of spermatozoan investigation, there also remains significant and rich scope for a host of statistical evaluation and analysis of even two-dimensional beating data, only recently available in appropriate quantities.

### 3.3. Towards Denser Populations

To date, the detailed study of flagellar kinematics has largely been restricted to lone individuals, with confounding factors present in both the imaging and analysis of multiple flagellated swimmers. This even carries over to theoretical study, where few works have considered multiswimmer settings in high fidelity (Walker et al., 2019b; Taketoshi et al., 2020). Indeed, only small numbers of swimmers, as few as two, are able to be considered without severe simplification. With crowded microenvironments being the norm for many spermatozoa, the next generation of experimental and theoretical analyses should seek to extend our multiswimmer understanding, from interrogating the details of polyswimmer synchrony, as observed by Woolley et al. (2009), to complex population-level interactions. The significant noted advances in both imaging techniques and digital processing will facilitate such developments, with the potential to generate unmatched quantities of data to drive investigative and exploratory study.

Another avenue for promising development is that of coarse-grained dynamics. Whilst the details of cellular geometry and flagellar beating can lead to distinct behaviours at the level of the individual swimmer, it is unclear how such effects scale up to populations. The approach of coarse graining, exemplified in Figure 2B for fluid flow but potentially applicable to other aspects of the problem, such as cell-cell interactions, may represent a viable method for capturing individual effects and thereby scaling up recent computational works, translating microscale mechanisms to complex multiswimmer environments.

### 3.4. Integrated Approaches

When considered in isolation, recent advances in imaging techniques and mathematical methods have each opened up new directions for exploring the world of a spermatozoon. Perhaps their most promising contribution, however, lies in their potential for synergy. In particular, the combination of high fidelity 3D imaging with modern hydrodynamic methods that afford efficiency, accuracy, and practical simplicity promises to lead to data-driven computational mechanical analyses in three dimensions, with similar explorations so far limited to two dimensions (Friedrich et al., 2010). In turn, this will enable the accurate and ready quantification of the forces and moments exerted on a beating flagellum, with the potential to further our understanding of the nature of active beating in spermatozoa.

Further, the advent of fast methods for the simulation of flagellar elastohydrodynamics brings with it the newfound possibility of realising parameter estimation in the context of swimming spermatozoa. In essence, parameter estimation techniques typically perform numerous simulations of a mathematical model, the results of which are then compared against data to provide refined estimates of model parameters and enable sophisticated model selection. Such methods can be impractical when the costs of simulation are high, much as they have previously been for flagellar elastohydrodynamics. Hence, with individual flagellar simulations having been sped up from many hours to only a few seconds (Moreau et al., 2018; Hall-McNair et al., 2019; Walker et al., 2019a, 2020a) or being performed in parallel (Larson et al., 2021), parameter estimation methods may now be readily applied in combination with datasets of spermatozoan beating, enabling the systematic calibration and assessment of computational models of the flagellum. In turn, this has the potential to be a rich new direction for quantitative analysis, informing biological enquiry and further refining the successful model-experiment cycle of spermatozoon study.

## 4. Summary

Our understanding of sperm motility mechanics has evolved dramatically since the inaugural studies of the 1950s, with extensive developments in the microscopy and data analysis of sperm swimming, together with advances in the theory and associated computational tools for flagellar beating in Newtonian fluids, as well as an elucidation of the underlying molecular motor mechanisms.

In the first part of this review, we introduced the mechanical interaction between the flagellum and the fluid, in particular the difference in the magnitude of the drag force perpendicular and parallel to the movement of the flagellum, which ultimately drives sperm swimming. More generally, we reviewed the spectrum of theories for flagellar-fluid mechanical interactions in Newtonian fluids, ranging from the simplest *resistive force theory* to the highly accurate but computationally expensive *boundary element methods*. We also surveyed the recent extensive gains in incorporating flagellar elastic responses within this framework, generating *elastohydrodynamic* models. In turn, these developments raise numerous opportunities for analysing the formation of the flagellar beat pattern and how it can be modulated by diverse features of the sperm microenvironment, such as background flows, chemoattractants, and confining geometries. The restriction to Newtonian media throughout the above also serves to emphasise the extensive need for developing the mechanics of sperm swimming in rheological fluids.

Concomitant to such theoretical developments, we have also highlighted the extensive progress that has been made in the digital imaging, video processing, and data analysis of the flagellar waveform. These advances have begun to allow population level data about the flagellum to be extracted from videomicroscopy, as well as high-resolution temporal and 3D spatial information. We have detailed how this presents many opportunities, for instance in using flagellar data for hypothesis testing at population levels, as well as raising a fundamental challenge for the field in parameter estimation and in integrating the many recent and diverse advances, both theoretical and observational, to further our understanding of the sperm flagellum. In summary, our survey has highlighted that the past 70 years of astonishing progress in the mechanics of sperm motility still leaves an immature field, with numerous opportunities and challenges remaining at the interfaces of applied mathematics, physics, and sperm cell and systems biology for the next 70 years.

## Author Contributions

EG, KI, and BW designed, drafted, and revised this review. All authors contributed to the article and approved the submitted version.

## Conflict of Interest

The authors declare that the research was conducted in the absence of any commercial or financial relationships that could be construed as a potential conflict of interest.
